# Circular Regression in a Dual-Phase Lock-In Amplifier for Coherent Detection of Weak Signal

**DOI:** 10.3390/s17112615

**Published:** 2017-11-14

**Authors:** Gaoxuan Wang, Serge Reboul, Jean-Bernard Choquel, Eric Fertein, Weidong Chen

**Affiliations:** 1 Laboratoire de Physico-Chimie de l’Atmosphère, Université du Littoral Côte d’Opale, 59140 Dnkerque, France; gaoxuanwang@gmail.com (G.W.); fertein@univ-littoral.fr (E.F.); 2 Laboratoire d’Informatique, Signal et Image de la Côte d’Opale (LISIC), Université du Littoral Côte d’Opale, 62228 Calais, France; Jean-Bernard.Choquel@univ-littoral.fr

**Keywords:** weak signal detection, lock-in amplifier, signal processing, circular regression, laser photoacoustic spectroscopy, trace gas detection

## Abstract

Lock-in amplification (LIA) is an effective approach for recovery of weak signal buried in noise. Determination of the input signal amplitude in a classical dual-phase LIA is based on incoherent detection which leads to a biased estimation at low signal-to-noise ratio. This article presents, for the first time to our knowledge, a new architecture of LIA involving phase estimation with a linear-circular regression for coherent detection. The proposed phase delay estimate, between the input signal and a reference, is defined as the maximum-likelihood of a set of observations distributed according to a von Mises distribution. In our implementation this maximum is obtained with a Newton Raphson algorithm. We show that the proposed LIA architecture provides an unbiased estimate of the input signal amplitude. Theoretical simulations with synthetic data demonstrate that the classical LIA estimates are biased for SNR of the input signal lower than −20 dB, while the proposed LIA is able to accurately recover the weak signal amplitude. The novel approach is applied to an optical sensor for accurate measurement of NO ${}_{2}$ concentrations at the sub-ppbv level in the atmosphere. Side-by-side intercomparison measurements with a commercial LIA (SR830, Stanford Research Inc., Sunnyvale, CA, USA ) demonstrate that the proposed LIA has an identical performance in terms of measurement accuracy and precision but with simplified hardware architecture.

## 1. Introduction

Lock-in amplifier (LIA) is an effective device capable of recovering weak signal buried in high noise level. This technique relies on locking an input signal to measure at a specific reference frequency. The signal is extracted from noise using phase sensitive detection (PSD) at this reference frequency [1,2]. In a LIA, the weak input signal is multiplied by a sine-wave signal that acts as reference signal [3]. This multiplication is processed with an analog multiplier, a digital switch or a digital multiplier [4]. A low-pass filter, with an appropriate cut-off frequency, is used to extract the mean value of the product between these two signals. The output filter value is proportional to the amplitude of the input signal and depends on a phase delay. This phase delay between the input signal and the sine-wave reference signal results from frequency fluctuations [5]. In this context it is important to set the reference phase to the value of the input phase signal in order to correctly recover the amplitude [6].

Phase locked loop (PLL) can be implemented in LIA to actively track the phase of the input signal. In this approach an internal reference generator is adjusted to match the phase of the input signal [7]. Andrea De Marcellis et al. [8] added a “phase alignment channel” and a “90 ${}^{\circ }$ phase shifter channel” to the LIA. These channels generate a reference in-phase signal and a quadrature signal with a 90 ${}^{\circ }$ shifted phase. The reference signal of the shifted channel and the input signal are provided to the PSD of the LIA. This novel LIA realizes an automatic phase alignment but needs, however, complex analog components and has a long response time.

Dual-phase LIA is an architecture widely used to remove the effects of the phase delay $\phi_{k}$ . In this architecture, two multiplications are performed with respectively a reference signal and the same signal in quadrature. A low pass filter is applied to the signals to produce the in-phase component $I=A\cos(\phi_{k})$ and the quadrature component $Q=A\sin(\phi_{k})$ . *A* is the amplitude of the input noisy signal, and $\phi_{k}=\arctan\left(Q/I\right)$ the phase delay. The in-phase and the quadrature components are squared and summed in order to remove the phase delay and to provide the input signal amplitude: $R=\sqrt{I^{2}+Q^{2}}=A$ [9]. However, *R* is a nonlinear function of *I* and *Q*. It can be shown that for the classical hypothesis of an additive Gaussian noise on the two components in quadrature, the random observations of *R* are distributed according to a Rician distribution [10]. When the signal-to-noise ratio (SNR) is high, the Rician distribution is similar to a Gaussian distribution and *A* can be estimated with observations of *R*. However, the Rician distribution deviates significantly from the Gaussian distribution at low SNR and the estimation of *A* with observations of *R* is biased. The bias is a function of the noise noise level [10].

**Figure 1:**
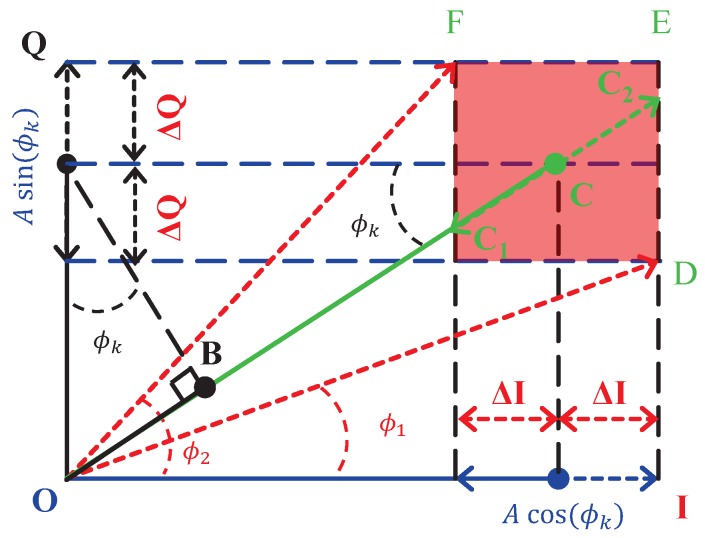
Principle of the determination of *R*. Abscissae represents the in phase component $I=A\cos\left(\phi_{k}\right)+\eta^{I}$ , and ordinate represents the quadrature component $Q=A\sin\left(\phi_{k}\right)+\eta^{Q}$ , where $\eta^{I}$ and $\eta^{Q}$ are Gaussian noise terms. $R=OC=\sqrt{I^{2}+Q^{2}}$ is an observation of *A* in conventional dual-phase LIA method. Let ΔI and ΔQ be the uncertainty in *I* and *Q*, caused by $\eta^{I}$ and $\eta^{Q}$ . $\phi_{1}$ and $\phi_{2}$ are the observed phase delays of the input noisy signal. The red area describes the uncertainties using traditional dual-phase LIA. In this article we consider the following observation $R^{c}=OB+BC=Q\sin\left(\phi_{k}\right)+I\cos\left(\phi_{k}\right)$ of *A*. In this case the area of uncertainties is a line between $C_{1}$ and $C_{2}$ . This area of uncertainties is smaller than in the classical case and it shows that the proposed observation $R^{c}$ is more accurate than *R*.

In the present work, we propose to determine the signal amplitude with observations $R^{c}=I\cos\left({\hat{\phi }}_{k}\right)+Q\sin\left({\hat{\phi }}_{k}\right)$ , where ${\hat{\phi }}_{k}$ is an estimate of $\phi_{k}$ . We show in Figure 1 that the observations $R^{c}$ are more accurate than the observations *R* provided by a classical coherent detection method. Furthermore we show in the next section that these observations are unbiased even at low SNR. This new approach requires, however, a precise estimate of the phase delay $\phi_{k}$ . This phase delay between the reference and the input signal is mainly due to frequency instability in the input noisy signal and is assumed to vary slowly [11]. It results in a linear phase delay variation. In this article, we introduce a linear-circular regression model to accurately estimate phase delays of the input noisy signal [12].

Signal processing of angular data (here the phase delay) relies on the modeling of angles in a circular Bayesian framework [13,14,15]. The noise is assumed to follow a circular normal von Mises distribution. This distribution acts in the circular domain as the normal distribution in the linear domain for observations defined on the real line [16,17]. Several nonlinear techniques have been proposed in the published works for angular data. These techniques include circular state estimation [18,19] and recursive filtering [20,21,22]. In the present work the circular regression of angular data is used to estimate the phase shift of the input signal [23].

The paper is organized as follows: the Section 2 describes the architecture of the proposed circular regression-based dual-phase digital LIA. The linear-circular model and method used to accurately estimate the phase delay are presented in the Section 3. In the Section 4, the proposed LIA is assessed using synthetic and real experimental data. Finally, high-sensitivity measurements of NO ${}_{2}$ trace gas in the atmosphere are presented and the performance of the proposed LIA is evaluated in comparison with a commercial LIA (SR830, Stanford Research Inc.).

## 2. Digital Signal Detection

**Figure 2:**
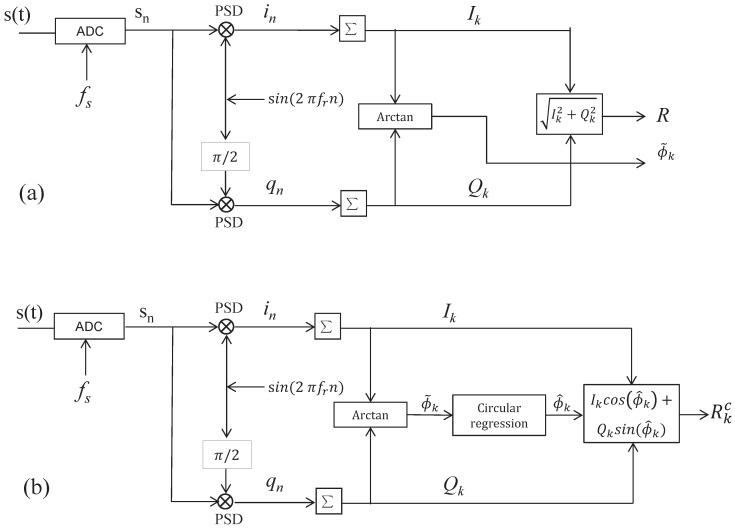
(**a**) Basic dual-phase lock-in amplification (LIA); (**b**) Linear-Circular regression-based dual-phase LIA. ADC: analog-digital convertor. ∑: sum function working as low-pass filter. Arctan: the inverse tangent function.

A typical dual-phase LIA (Figure 2a) and the proposed circular regression-based dual-phase LIA (Figure 2b) are schematically depicted in Figure 2. We assume that an input signal s(t) contains a carrier of amplitude *A* at a frequency *f*. This signal is digitized at a sampling frequency $f_{s}$ by an analog–to-digital convertor (ADC). This discrete sampled signal $s_{n}$ can be expressed as a function of the sampling period $T_{s}$ with the following expression:

(1) $$\begin{array}{c} s_{n}=A\;sin\left(2\pi fnT_{s}\right)+\eta_{n} \end{array}$$

where $\eta_{n}$ is a Gaussian noise. The general aim of lock-in detection is to extract the amplitude value *A*, buried in noise. We define the SNR of the input signal as follows:

(2) $$\begin{array}{c} SNR=\frac{A}{\sigma_{g}} \end{array}$$

where $\sigma_{g}$ is the standard deviation of the Gaussian noise $\eta_{n}$ . The SNR in dB is:

(3) $$\begin{array}{c} SNR_{dB}=10\log_{10}\left(\frac{A^{2}}{\sigma_{g}^{2}}\right) \end{array}$$

The in-phase $i_{n}$ component and the quadrature $q_{n}$ component are respectively defined as the multiplication of the input signal with the reference signal (at frequency $f_{r}$ ) and the multiplication of the input signal with the reference signal shifted by π/2 . $i_{n}$ and $q_{n}$ are expressed as follows:

(4) $$\begin{array}{ccc} i_{n} & = & \frac{A}{2}\left(\cos\left(2\pi \left(f_{r}-f\right)nT_{s}\right)+\cos\left(2\pi \left(f_{r}+f\right)nT_{s}\right)\right)+\eta_{n}^{i} \end{array}$$

(5) $$\begin{array}{ccc} q_{n} & = & \frac{A}{2}\left(\sin\left(2\pi \left(f_{r}-f\right)nT_{s}\right)+\sin\left(2\pi \left(f_{r}+f\right)nT_{s}\right)\right)+\eta_{n}^{q} \end{array}$$

where $\eta_{n}^{i}$ and $\eta_{n}^{q}$ are independent Gaussian noises with a variance of $\sigma_{g}^{2}/2$ . In order to filter out the frequency component $f_{r}+f$ , $T_{w}f_{s}$ samples of the signals $i_{n}$ and $q_{n}$ are summed on a working window $T_{w}$ . This sum acts as a low-pass filter. The integration time $T_{w}$ corresponds to the time constant in classic dual-phase LIA (Figure 2a). The choice of $T_{w}$ will be investigated in a next section (Section 4.3). Two components values, $I_{k}$ and $Q_{k}$ , are processed at each time instant *k*, multiple of the period $T_{w}$ . Expressions of the two components after integration by the low-pass filter are given below:

(6) $$\begin{array}{c} I_{k}=\frac{Af_{s}T_{w}}{2}\cos\left(\phi_{k}\right)+\eta_{k}^{I} \end{array}$$

(7) $$\begin{array}{c} Q_{k}=\frac{Af_{s}T_{w}}{2}\sin\left(\phi_{k}\right)+\eta_{k}^{Q} \end{array}$$

where $\eta_{k}^{I}$ and $\eta_{k}^{Q}$ are Gaussian noises with a variance of $f_{s}T_{w}\sigma_{g}^{2}/2$ . $\phi_{k}$ is the phase delay between the input signal and the reference. This phase delay evolves linearly with time when the frequency difference $f_{r}-f$ is constant. In a classical dual-phase LIA (Figure 2a), the noisy observations of $I_{k}$ and $Q_{k}$ are processed with the following expressions to observe noisy values of the signal amplitude and the phase delay:

(8) $$\begin{array}{ccc} R & = & \sqrt{I_{k}^{2}+Q_{k}^{2}} \end{array}$$

(9) $$\begin{array}{ccc} {\tilde{\phi }}_{k} & = & \arctan\frac{Q_{k}}{I_{k}}=\phi_{k}+\Delta \phi_{k} \end{array}$$

where $\Delta \phi_{k}$ is the phase noise related to Gaussian noises $\eta_{k}^{I}$ and $\eta_{k}^{Q}$ . Figure 2b shows the proposed LIA architecture. In this architecture, we notice ${\hat{\phi }}_{k}$ the phase delay obtained with the linear-circular regression estimator. With the proposed approach, noisy observations of $I_{k}$ and $Q_{k}$ are processed with the following expression to observe the signal amplitude:

(10) $$\begin{array}{c} R_{k}^{c}=I_{k}\cos\left({\hat{\phi }}_{k}\right)+Q_{k}\sin\left({\hat{\phi }}_{k}\right) \end{array}$$

With a linear-circular regression approach the estimated phase delay ${\hat{\phi }}_{k}$ is between ${\tilde{\phi }}_{k}$ and $\phi_{k}$ . The estimate ${\hat{\phi }}_{k}$ tends to $\phi_{k}$ when the number of observations used to process the circular estimate is high. In the opposite, the estimate ${\hat{\phi }}_{k}$ tends to ${\tilde{\phi }}_{k}$ when the number of observations is low. The corresponding expressions for $R_{k}^{c}$ are given below.

- When ${\hat{\phi }}_{k}={\tilde{\phi }}_{k}$
(11)Rkc=Ikcos(ϕ˜k)+Qksin(ϕ˜k)(12)=IkIkIk2+Qk2+QkQkIk2+Qk2(13)=Ik2+Qk2=AfsTw2+ηkC
  where $\eta_{k}^{C}$ is a Rician noise. In this case, $R_{k}^{c}=R_{k}$ and the proposed approach is equivalent to the classical dual-phase approach. The noise on the observed signal is distributed according to a Rician distribution. This approach is called incoherent detection (ID) in the rest of the article.
- When ${\hat{\phi }}_{k}=\phi_{k}$
(14)Rkc=Ikcos(ϕk)+Qksin(ϕk)(15)=AfsTw2cos2ϕk+AfsTw2sin2ϕk+ηkIcos2ϕk+ηkQsin2ϕk(16)=AfsTw2+ηkC
  In this case, $\eta_{k}^{C}=\eta_{k}^{I}\cos^{2}\left(\phi_{k}\right)+\eta_{k}^{Q}\sin^{2}\left(\phi_{k}\right)$ is a Gaussian noise. This is the proposed approach. In this case the noise on the observed signal amplitude is distributed according to a Gaussian distribution. This approach is called coherent detection (CD).

**Figure 3:**
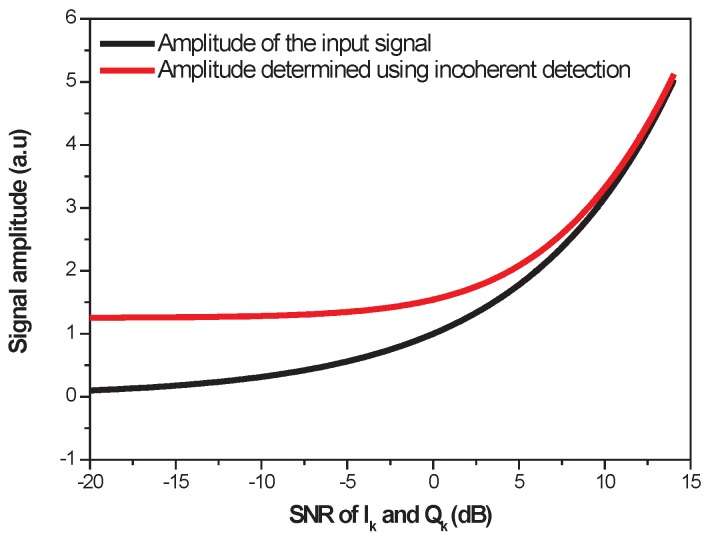
Mean estimate of A in the case of incoherent detection (ID).

We show in Figure 3 the mean value of *A* obtained with observations of $R_{k}^{c}$ . The x-axis is the SNR of the components $I_{k}$ and $Q_{k}$ . The black line shows the values of the input signal amplitude *A*. The black line is also the mean value of *A* obtained with the CD method for a Gaussian noise on the observations. The red line represents the mean value of *A* obtained with the ID method for a Rician noise on the observations. These curves show that the ID method (incoherent detection) is biased in case of weak signal detection, especially when the SNR of the $I_{k}$ and $Q_{k}$ components is less than 5 dB. These results are obtained for a Rician probability distribution given by the following equation:

(17) $$\begin{array}{c} f\left(x|A,\sigma \right)=\frac{x}{\sigma^{2}}\exp\left(\frac{-x^{2}-A^{2}}{2\sigma^{2}}\right)I_{0}\left(\frac{x\;A}{\sigma^{2}}\right) \end{array}$$

where $\sigma^{2}$ is the variance of the Gauss noises $\eta_{k}^{I}$ and $\eta_{k}^{Q}$ . $I_{0}$ is the modified Bessel function of first kind and order zero. The mean value μ of the Rician distribution is obtained with the following expression:

(18) $$\begin{array}{c} \mu =\sigma \sqrt{\frac{\pi }{2}}L_{1/2}\left(\frac{-A^{2}}{2\sigma^{2}}\right) \end{array}$$

where $L_{q}\left(x\right)$ is the Laguerre polynomial.

## 3. Circular Regression

Circular regression has been used in several applications such as wind and wave direction estimation [23,24], GNSS altimetry [25]... In this article we use a linear-circular regression to estimate the phase delay between a reference signal and the signal to be measured. In this model the abscissae is linear and the ordinate is an angle. We can find in the published works three different circular regressions; the circular-linear regression, the circular-circular regression and the linear-circular regression [16].

The main difficulty in a linear-circular regression is the circular nature of the variable response. One way to address the circularity is to assume that the noise is distributed according to a von Mises distribution. We can find in the published works different maximum likelihood approaches for the regression parameters estimation [26,27,28,29]. In this article we derived a similarity function from the likelihood and we propose to maximize this similarity function with a Newton Raphson algorithm.

In this section, we derive an accurate estimate of the phase delay $\phi_{k}$ of the input signal. We consider a working window $T_{p}$ and observations obtained with a period $T_{w}$ . The number of observations used to process the linear-circular regression thus is $n=T_{p}/T_{w}$ . The choice of the working window $T_{p}$ is discussed in the next section, dedicated to simulation assessment of the proposed LIA approach. The observed phase delay ${\tilde{\phi }}_{k}$ is a circular variable which takes its values on the circumference of a circle. This variable is assumed to follow a linear-circular model with an additive angular noise following a circular von Mises distribution. The noisy linear-circular model is described as follows:

(19) $$\begin{array}{c} y_{k}=\alpha +\beta x_{k}+\epsilon_{k}\left(mod\;2\pi \right) \end{array}$$

where $y_{k}$ models the observations of the phase delay ${\tilde{\phi }}_{k}$ at $x_{k}$ . $\epsilon_{k}$ is a zero-mean noise distributed according to a von Mises distribution with concentration parameter κ . α and β are the y-intercept and slope of the linear-circular regression. In our application the slope is equal to $\beta =2\pi (f_{r}-f)$ . mod2π is the modulo of 2π . The von Mises distribution is defined by the following equation:

(20) $$\begin{array}{c} f\left(y_{k}/\alpha ,\beta ,\kappa \right)=\frac{1}{2\pi I_{0}\left(\kappa \right)}exp\left(\kappa \cos\left(y_{k}-\left(\alpha +\beta x_{k}\right)\right)\right) \end{array}$$

The likelihood of n samples $y_{k}$ is defined by:

(21) $$\begin{array}{c} L\left(y_{1},\ldots ,y_{n}\right)=f\left(y_{1}/\alpha ,\beta ,\kappa \right)\ldots f\left(y_{n}/\alpha ,\beta ,\kappa \right) \end{array}$$

The likelihood of n samples for the von Mises distribution is given by:

(22) $$\begin{array}{c} L\left(y_{1},\ldots ,y_{n}\right)=\frac{1}{{\left(2\pi I_{0}\left(\kappa \right)\right)}^{n}}exp\left(\kappa \sum_{k=1}^{n}\cos\left(y_{k}-\left(\alpha +\beta x_{k}\right)\right)\right) \end{array}$$

The log-likelihood function is given by:

(23) $$\begin{array}{c} l\left(y_{1},\ldots ,y_{n}\right)=\log\left(L(y_{1},\ldots ,y_{n})\right)\\ =\;\;-n\log\left(2\pi I_{0}\left(\kappa \right)\right)+\;\kappa \sum_{k=1}^{n}\cos\left(y_{k}-\left(\alpha +\beta x_{k}\right)\right) \end{array}$$

In order to derivate the maximum likelihood estimate $\hat{\alpha }$ of α , the log-likelihood function is derived with respect to α , and the result is given in Equation (24):

(24) $$\begin{array}{c} \frac{\partial l(y_{1},\ldots ,y_{n})}{\partial \alpha }=\kappa \sum_{k=1}^{n}\sin\left(y_{k}-\left(\alpha +\beta x_{k}\right)\right) \end{array}$$

Equation (24) is set to zero to obtain the estimate of α :

(25) $$\begin{array}{ccc} \frac{\partial l(y_{1},\ldots ,y_{n})}{\partial \alpha } & = & 0\\ \cos\left(\alpha \right)\sum_{k=1}^{n}\sin\left(y_{k}-\beta x_{k}\right) & = & \sin\left(\alpha \right)\sum_{k=1}^{n}\cos\left(y_{k}-\beta x_{k}\right) \end{array}$$

Finally the estimate of α is obtained:

(26) $$\begin{array}{c} \hat{\alpha }=\arctan^{*}\left(\frac{\sum_{k=1}^{n}\sin\left(y_{k}-\beta x_{k}\right)}{\sum_{k=1}^{n}\cos\left(y_{k}-\beta x_{k}\right)}\right) \end{array}$$

where $Arctan^{*}\left(\ldots \right)$ is the “quadrant-specific” inverse of the tangent. It is shown in [19] that $Arctan^{*}\left(\ldots \right)$ is the maximum likelihood estimate. In order to compute the maximum likelihood estimate $\hat{\beta }$ of β , the 1st and 2nd order derivatives of the log-likelihood with respect to β are calculated:

(27) $$\begin{array}{ccc} \frac{\partial l(y_{1},\ldots ,y_{n})}{\partial \beta } & = & \kappa \sum_{k=1}^{n}x_{k}\sin\left(y_{k}-\left(\alpha +\beta x_{k}\right)\right) \end{array}$$

(28) $$\begin{array}{ccc} \frac{\partial^{2}l\left(y_{1},\ldots ,y_{n}\right)}{\partial \beta^{2}} & = & -\kappa \sum_{k=1}^{n}x_{k}^{2}\cos\left(y_{k}-\left(\alpha +\beta x_{k}\right)\right) \end{array}$$

In this context the second order Taylor approximation of l(…) at $y_{1},\ldots ,y_{n}$ can be obtained with the Newton Raphson algorithm and the values of α and β that maximize the log-likelihood are recursively obtained by:

(29) $$\begin{array}{ccc} \alpha_{i} & = & \arctan^{*}\left(\frac{\sum_{k=1}^{n}\sin\left(y_{k}-\beta_{i-1}x_{k}\right)}{\sum_{k=1}^{n}\cos\left(y_{k}-\beta_{i-1}x_{k}\right)}\right) \end{array}$$

(30) $$\begin{array}{ccc} \beta_{i} & = & \beta_{i-1}+\frac{1}{2}\frac{\sum_{k=1}^{n}x_{k}\sin\left(y_{k}-\left(\alpha_{i}+\beta_{i-1}x_{k}\right)\right)}{\sum_{k=1}^{n}x_{k}^{2}\cos\left(y_{k}-\left(\alpha_{i}+\beta_{i-1}x_{k}\right)\right)} \end{array}$$

$\alpha_{i}$ and $\beta_{i}$ converge to the maximum likelihood estimates $\hat{\alpha }$ and $\hat{\beta }$ . The circular regression is equivalent to the linear regression when $y_{k}$ is unwrapped. In this context the variance of the estimate $\hat{\beta }$ is defined by:

(31) $$\begin{array}{c} var\left(\hat{\beta }\right)=\frac{\sigma_{l}^{2}}{\sum_{k=1}^{n}{\left(kT_{w}-\bar{t}\right)}^{2}} \end{array}$$

The link between the standard deviation $\sigma_{l}$ in the linear domain and the parameter of concentration κ in the circular domain is defined by [17]:

(32) $$\begin{array}{c} \sigma_{l}^{2}=-2\ln\left(A\left(\kappa \right)\right) \end{array}$$

(33) $$\begin{array}{c} \mathrm{with}A\left(\kappa \right)=\frac{I_{1}\left(\kappa \right)}{I_{0}\left(\kappa \right)} \end{array}$$

where $I_{n}\left(\ldots \right)$ is the modified Bessel function of the first kind of order n. Finally the relation between κ and the SNR of s(t) can be approximated using the following linear expression:

(34) $$\begin{array}{c} \kappa =\frac{f_{s}T_{w}}{2}\;SNR \end{array}$$

## 4. Experimental Assessments

In order to characterize the ID and CD approaches and to optimize the typical parameters such as $T_{w},T_{p}$ , synthetic data are simulated with MATLAB in a first step. In a second step, the proposed CD approach is applied to an optical NO ${}_{2}$ sensor for experimental validation.

### 4.1. Simulation Assessment Using Synthetic Data

**Figure 4:**
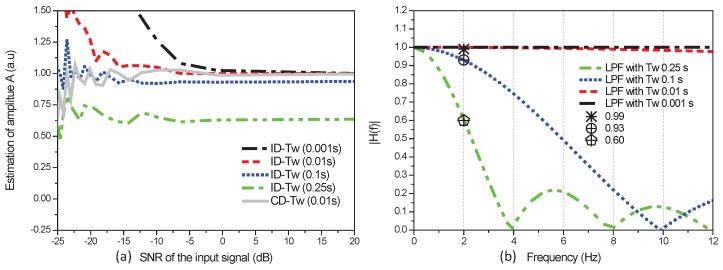
(**a**) Estimates of the input signal amplitude *A* as a function of the signal-to-noise ratio (SNR). Assessment of coherent detection (CD) and incoherent detection (ID) methods for different $T_{w}$ : 0.001 s, 0.01 s, 0.1 s and 0.25 s; (**b**) Frequency response of the Low-Pass Filters (LPF) with $T_{w}$ of 0.001 s, 0.01 s, 0.1 s and 0.25 s.

The ID and CD methods are assessed through MATLAB simulations. We show in Figure 4a the ID and CD estimates of the input signal amplitude *A*. These estimates are plotted in Figure 4a as a function of the SNR. The amplitude *A* is fixed to 1.0 V. The difference of frequency between the signal and the local reference is $f_{r}-f$ = 2 Hz (a typical frequency shift of a mechanical chopper usually used for amplitude modulation in optical sensors [11]). The linear-circular regression is processed with phase delay observations obtained in a working window $T_{p}$ = 1 s. The sampling frequency is $f_{s}$ = 44 kHz. The observations of *A* provided by the ID and CD methods are averaged within an observation window $T_{o}$ = 1 s to provide an estimate $\hat{A}$ :

(35) $$\begin{array}{c} \hat{A}=\frac{T_{w}}{T_{o}}\sum_{k=1}^{\frac{T_{o}}{T_{w}}}A_{k} \end{array}$$

As can be seen in Figure 4a, for weak signals with a SNR lower than −5 dB, the ID estimate deviates significantly from the real signal amplitude when the integration period is $T_{w}$ = 0.001 s (black line) or $T_{w}$ = 0.01 s (red line). This effect is due to the biased observations provided by the ID method. For a period $T_{w}$ = 0.01 s (grey line), the CD estimate does not deviate significantly from the real signal amplitude even for a SNR lower than −5 dB. In this case, the observations provided by the CD method are indeed unbiased.

In order to improve the ID estimate and to remove the bias, the integration period $T_{w}$ is increased. In this case we can observe in Figure 4a that the amplitude is underestimated for $T_{w}$ = 0.1 s (blue line) or $T_{w}$ = 0.25 s (green line). This underestimation is explained in Figure 4b with the study of the lock-in detection process in the frequency domain.

In lock-in detection, the incoming signal is down converted at frequency $f_{r}-f$ and then filtered out by a low-pass filter (sum function). The transfer function of the low pass filter is given by the following equation:

(36) $$\begin{array}{c} H\left(f\right)=\frac{\sin\left(\pi fNT_{s}\right)}{\sin\left(\pi fT_{s}\right)}\exp\left(-j2\pi f\left(N-1\right)T_{s}\right) \end{array}$$

where $N=f_{s}T_{w}$ , is the number of observations in an integration period $T_{w}$ . Figure 4b shows the transfer function of low-pass filters with different integration periods . As $f_{r}-f$ = 2 Hz, the amplitude output of the low-pass filter with integration periods 0.25 s, 0.1 s, 0.01 s and 0.001 s are respectively 0.60, 0.93, 0.99 and 1. These amplitudes are close to the amplitude estimated with the ID method and it demonstrates that a period $T_{w}$ larger than 0.25 s results in an underestimated signal amplitude with a bias ratio of 0.60. In addition, larger $T_{w}$ results in a longer response time of the LIA detection. As a conclusion, using the ID method, the amplitude is either biased at low signal to noise ratio (for large $T_{o}$ ) or underestimated at any signal to noise ratio (for large $T_{w}$ ). In this context, the only way to improve the estimation process is to estimate the signal amplitude with CD observations processed with long observation window $T_{o}$ and short integration time $T_{w}$ .

As a conclusion, using the ID method, the amplitude is either biased at low signal to noise ratio (for large $T_{o}$ ) or underestimated at any signal to noise ratio (for large $T_{w}$ ).

The amplitude observation window $T_{o}$ influences the performance of the CD approach for recovery of weak signals amplitude. We show in Table 1 the minimum SNR required for the estimation of the input signal amplitude with an uncertainty lower than 10% . In this simulation, the integration time $T_{w}$ is set to 10 ms, $T_{p}$ = 1 s and the frequency difference is fixed to 2 Hz. The minimum SNR of the input signal varies from −14 dB to −22 dB when $T_{o}$ varies from 0.25 s to 1 s. It tends to be constant (−24 dB) with a period $T_{o}$ larger than 2 s. Increasing the size of $T_{o}$ from 0.25 s to 1 s with a $T_{w}$ = 0.01 s can enhance the estimation accuracy, but for an observation window superior to 2 s the performance remains the same.

**Figure 5:**
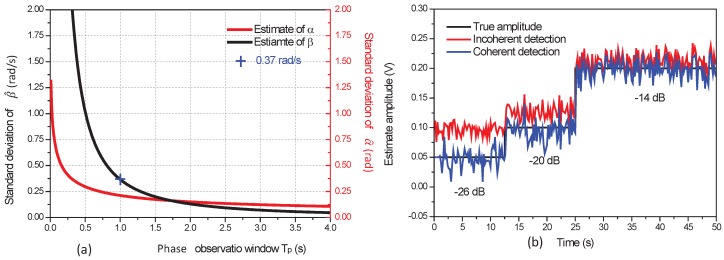
(**a**) Standard deviation of $\hat{\beta }$ and $\hat{\alpha }$ for a SNR of −22 dB; (**b**) Example of LIA detection using coherent and incoherent detections.

The estimation accuracy of the CD method can be enhanced with the phase observation window $T_{p}$ . The dependence of the standard deviation (SD) of the estimated slope (that reflects the frequency difference) over the phase observation window $T_{p}$ is investigated Figure 5a. We present in Figure 5a the standard deviation of the estimates $\hat{\alpha }$ and $\hat{\beta }$ as a function of the phase observation window $T_{p}$ for a SNR of −22 dB. We show in Figure 5a that the SD of $\hat{\alpha }$ and $\hat{\beta }$ decrease when the period of observation $T_{p}$ increases. In practice a period of observation $T_{p}$ = 1 s and an integration period $T_{w}=$ 10 ms are used. This choice is based on the simulations results. We indeed observe in Figure 5a that the SD of the estimated slope with $T_{p}$ = 1 s is 0.37 rad/s. This SD value corresponds to a frequency difference of 0.06 Hz which is sufficiently accurate for a typical frequency shift of 2 Hz.

Figure 5b shows a measurement example obtained with simulated data. The phase delay of the input signal is estimated with the circular regression of the CD method discussed in Section 3. For this experiment the amplitude *A* changes from 0.05 V to 0.10 V and to 0.20 V and the corresponding SNR of the input signals are, respectively, −26 dB, −20 dB and −14 dB. The difference of frequency between the signal and the local replica is fixed to $f_{l}-f$ = 2 Hz. For the CD and ID approaches, an integration time $T_{w}$ =10 ms and a phase observation window Tp = 1 s are applied. The signal amplitudes estimated by ID approach for a SNR of −26 dB, −20 dB, −14 dB are respectively 0.10±0.01 V, 0.13±0.01 V and 0.21±0.02 V. The signal amplitudes estimated by CD approach are 0.05±0.02 V, 0.10±0.02 V and 0.20±0.02 V and are well consistent with the true values. As expected, the estimated signal amplitudes with the ID approach are biased for a SNR less than −20 dB. The same conclusions are observed with LIA detection of a real signal, as presented in the next paragraph.

### 4.2. Application to High-Sensitivity and Accurate Measurements of NO ${}_{2}$ at ppbv Concentration Level in the Environment

We implement the proposed “circular phase processing” LIA approach in an optical sensor for accurate measurements of low trace gas concentration of NO ${}_{2}$ in the atmosphere. The optical sensor is based on photoacoustic spectroscopy (PAS). In this sensor a pressure wave (acoustic signal) is generated by thermal expansion resulting from absorption of a power-modulated light by target gas [30]. The resulting acoustic signal, proportional to the target gas concentration, is detected with microphones.

**Figure 6:**
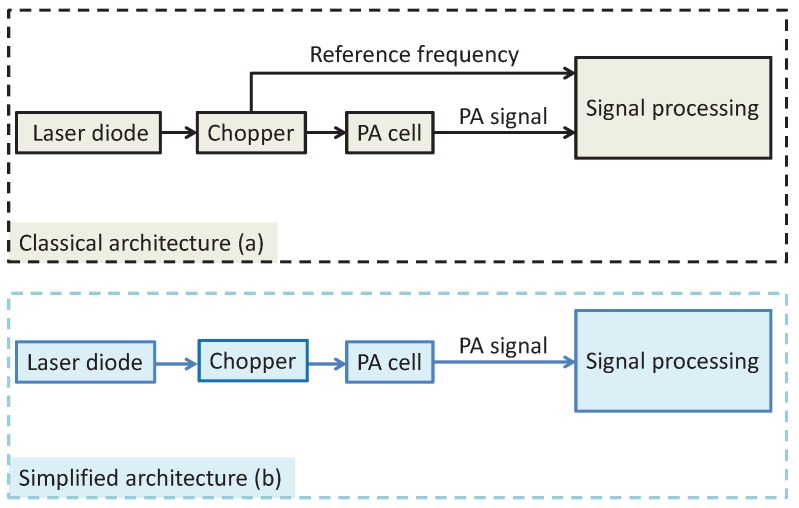
Architectures of LIA involved in a PAS-based NO ${}_{2}$ sensor.

Figure 6a describes a classical LIA architecture of a PAS sensor. A laser beam, modulated with a mechanic chopper at a “reference frequency”, is used to probe absorption of NO ${}_{2}$ . The reference frequency is then used to demodulate the signal with a traditional lock in amplifier. The photoacoustic (PA) cell is a single-pass cylinder which has a length of 26 mm and a diameter of 6 mm [31]. The proposed LIA architecture is described in Figure 6b. The proposed approach uses a fix frequency and no tracking of the reference frequency is necessary.

**Figure 7:**
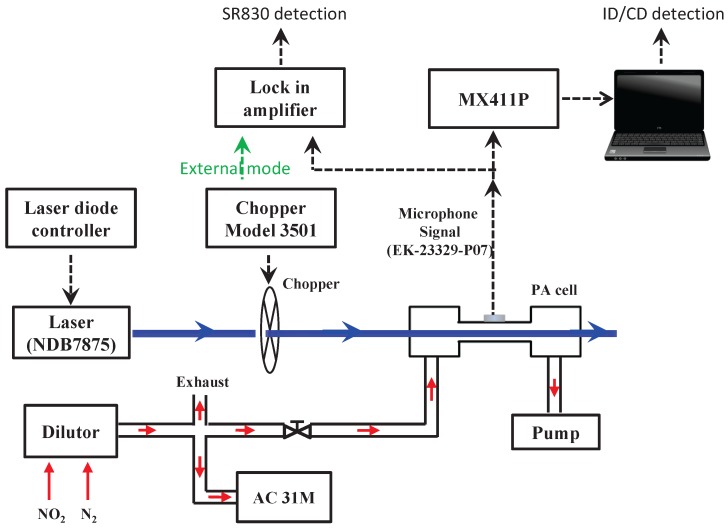
Experimental set up of photoacoustic spectroscopy (PAS)-based NO ${}_{2}$ sensor.

The detailed experimental set-up is shown in Figure 7. A high power laser diode (NDB7875, Nichia, Tokushima, JAPAN) operating at a wavelength of 444 nm is used as light source. The laser light, driven by a laser diode controller (6340, Arryo instrument, San Luis Obispo, CA, USA), is amplitude-modulated using a mechanical chopper (3501, New Focus, Inc., San Jose, CA, USA). The modulation frequency is matched to the fundamental longitudinal resonance frequency of the cylinder (in order to be an acoustic resonator) at 6260 Hz. Electric condenser microphones (EK-23329-P07, Knowels, Itasca, IL, USA) are used to record the acoustic signal in the PA cell.

Photoacoustic signals are sampled and digitized with a data acquisition system (MX411-P, HBM, Darmstadt, Germany). The following configurations are adopted: a 24-bit ADC at a sampling rate of 96 kHz. As a reference instrument used for inter-comparison, a classical dual-phase LIA SR830 (Stanford Research Inc.) is used with the following configuration: 16-bit DAC and a sampling rate of 256 kHz. The proposed CD approach is implemented in real time with MATLAB.

For NO ${}_{2}$ concentration calibration, we use a standard reference concentration of 10 ppmv (parts per million by volume) NO ${}_{2}$ , diluted with pure nitrogen (N ${}_{2}$ ). We obtain different NO ${}_{2}$ concentrations varying from 6 ppbv to 100 ppbv (parts per billion by volume) using a dilutor (Modele PPA 2000M). The photoacoustic signal is calibrated with measurement of NO ${}_{2}$ concentration provided by an analyzer (AC-31M). The ID and the proposed CD approaches are used to measure different NO ${}_{2}$ concentrations.

**Figure 8:**
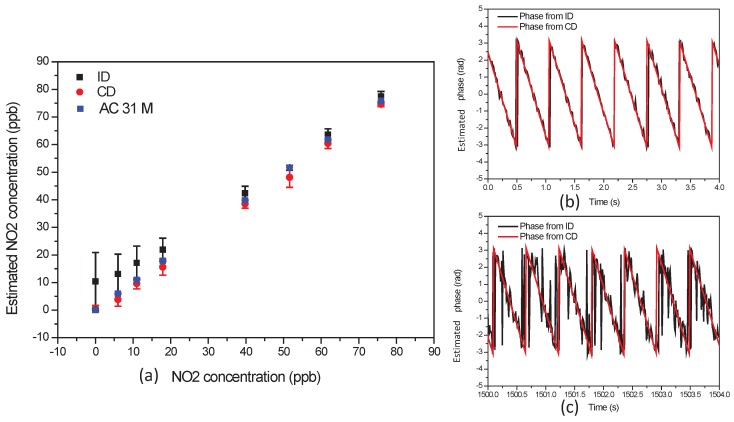
(**a**) Estimated NO ${}_{2}$ concentrations using ID and CD approaches with regards to the NO ${}_{2}$ concentration measured by AC-31M analyzer. The error bars are the root mean square error between the AC-31M measurements and the estimates obtained with the ID or CD methods; (**b**) Observed and estimated phase delay for a NO ${}_{2}$ concentration of 76 ppbv; (**c**) Observed and estimated phase delay for a NO ${}_{2}$ concentration of 6 ppbv.

Figure 8a shows the estimated NO ${}_{2}$ concentrations obtained with the ID and CD methods as a function of the NO ${}_{2}$ concentration measured by the NOx analyzer AC-31M. For this experimentation the period of observation of the phase delay is $T_{p}$ = 1 s. For both ID and CD measurements the integration time is $T_{w}$ = 0.01 s. The estimated concentration is the average of the measurements obtained over a period $T_{o}$ = 1 s. In Figure 8a the error bar is the root mean square error between the NO ${}_{2}$ concentration measured by the AC-31M analyzer and the concentration estimates obtained with the CD and ID methods. At low concentration, the error bars of the estimates obtained with the ID method are larger than the error bar of the estimates obtained with the CD approach. The inaccuracy of the ID approach in this case is due to the estimation bias of the method. It is obvious that the ID approach can not accurately recover NO ${}_{2}$ concentration less than 20 ppbv. The CD method overcomes this limitation. These results are in accordance with the previously presented theoretical study and assessment using synthetic data.

Figure 8b,c show the observed and estimated phase delay for a NO ${}_{2}$ concentration of respectively 76 ppbv and 6 ppbv. We can observe in Figure 8b,c that the observations of phase delay are more noisy when the concentration of NO ${}_{2}$ decreases. We can observe in Figure 8c that the linear-circular regression is not affected by the 2π transitions due to the periodic nature of the angular observations. Figure 8b,c, the cycle of the observed phase is 0.56 s, which corresponds to a difference frequency $\left(f_{r}-f\right)$ of 1.79 Hz and an estimated slope $\hat{\beta }$ of −11.24 rad/s.

### 4.3. Comparison Measurements with Commercial LIA

An inter-comparison of NO ${}_{2}$ measurement at low concentrations is carried out using a commercial LIA (SR830) and the proposed LIA architecture. In order to assess the proposed LIA architecture, the SR830 LIA is implemented in two different operation modes. In the first implementation (called external mode SR830E), a link between the chopper and the LIA (as shown in Figure 7, the green arrow) provides an accurate measurement of the PA signal frequency. In the second implementation using an internal mode SR830I, the reference frequency is fixed to 6260 Hz and the above link is removed. These two implementations are compared with the proposed CD method when the integration time is increased.

**Figure 9:**
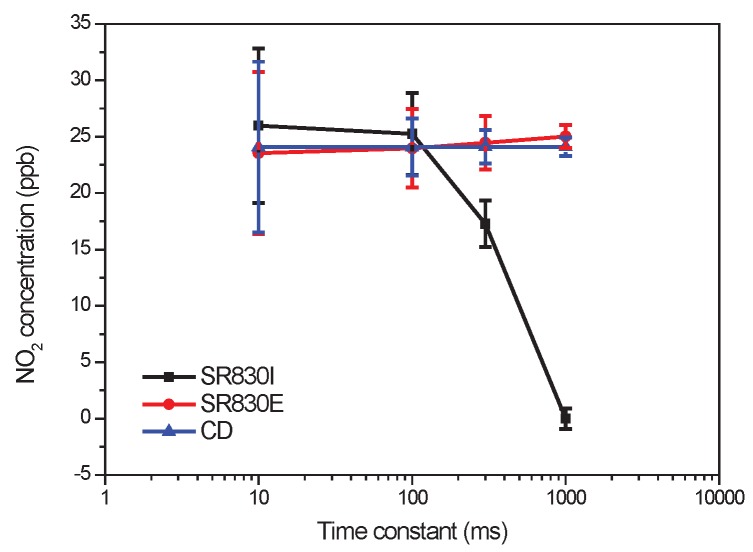
Performance comparisons of the estimated NO ${}_{2}$ concentration obtained with a SR830 lock-in amplifier and the proposed CD approach. The error bars are the standard deviation of the concentration estimates.

Figure 9 shows the experimental results for the measurement of a NO ${}_{2}$ concentration of 24.5 ppbv. We show in Figure 9 the assessments of the three different methods and several time constants. The mean value and the standard deviation (error bars) of each estimate are processed in an experimentation of 60 s. In this experiment the time constant Figure 9 is the period $T_{w}$ for SR830E and SR830I LIA. For CD implementation $T_{w}$ is fixed to 0.01 s, $T_{p}$ is fixed to 1 s and the time constant (the abscissa Figure 9) is the period $T_{o}$ .

The results presented in Figure 9 show that the LIA SR830I in internal mode underestimates the concentration when the time constant is >100 ms. This underestimation is due to the frequency difference between the reference frequency and the modulation frequency of the mechanic chopper.

**Figure 10:**
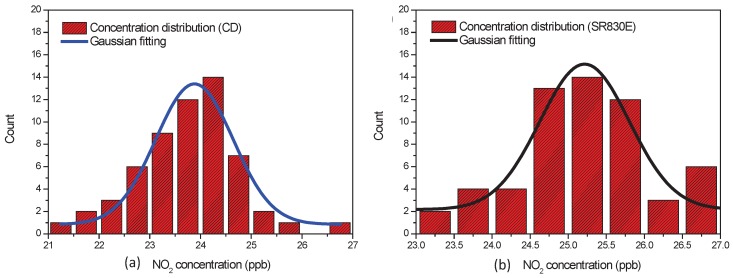
Histogram showing the distribution of the estimated NO ${}_{2}$ concentration (of 24.5 ppbv measured with a reference NOx analyser) by CD (**a**) and SR830E (**b**).

Figure 10a,b show the distribution histogram of the estimated NO ${}_{2}$ concentrations by CD and SR830E LIA with a time constant of 1000 ms. The distribution histograms of the measured concentration are fitted to a Gaussian profile. The mean concentration of 23.9 ppbv obtained by CD method results in an accuracy of 0.6 ppbv (Figure 10a) with a precision of 1.8 ppbv. The mean concentration obtained by the SR830E LIA shown in Figure 10b is 25.2 pppv. Its measurement accuracy and precision are found to be 0.7 ppbv and 1.4 ppbv, respectively.

As can be seen in Figure 9 and Figure 10, the proposed CD method and the LIA SR830E working in external mode have close performance. The advantage of the proposed CD method is that the linear-circular regression accurately estimates the phase delay and removes the noisy phase fluctuations. The implementation of the proposed CD approach is easier and more robust because it only requires a fix reference frequency and simplified hardware components, while SR830 adopts complicated hardware such as low noise amplifier and notch filter to reduce the noise in the input signal and a phase locked loop to track the phase delay.

## 5. Conclusions

We introduce in the present work a new dual-phase Lock-In Amplification (LIA) processing for coherent detection. The proposed approach relies on precise estimation of the phase delay between the input signal and a reference signal. In our implementation we propose a linear-circular regression estimate of the phase delay. We show with theoretical results and synthetic experimentation that the proposed coherent detection estimate is more accurate than the classical incoherent detection estimate. We show that the proposed approach accurately recovers weak signal intensity at low SNR and outperforms the classical incoherent detection approach for a SNR of the input signal lower than −20 dB.

Coherent detection and incoherent detection approaches are then applied to a PAS-based optical sensor for NO ${}_{2}$ concentration measurements in the atmosphere. The estimated concentrations are assessed with a reference instrument of NO ${}_{2}$ analyzer. The measurements carried out at different NO ${}_{2}$ concentrations indicate that the estimated concentration from incoherent detection approach is biased when NO ${}_{2}$ concentration is less than 20 ppbv. We show that the proposed coherent detection approach overcomes this limitation. This result is in accordance with the theoretical study and assessment using synthetic data.

The measurements comparison, for a constant NO ${}_{2}$ concentration of 24.5 ppbv and different time constants of integration, indicates that the proposed coherent detection LIA and the commercial LIA SR830 have close performances in terms of measurement accuracy and precision. However, the proposed LIA architecture is simpler and the processing is more robust. In future work, the proposed LIA approach using linear-circular regression will be implemented in a field programmable gate array for atmospheric measurements of trace gases pollutants.

## Figures and Tables

**Table 1:** Minimum SNR obtained for the estimation of the input signal amplitude with an uncertainty less than 10% . The SNR is provided as a function of the observation window $T_{o}$ .

$T_{o}$ **(s)**	0.25	0.50	1.00	1.50	2.00	2.50	3.00	3.50	4.00
**SNR (dB)**	−14	−19	−22	−23	−24	−24	−24	−24	−24
